# Functional Foods and Sustainable Health (2nd Edition)

**DOI:** 10.3390/nu18121864

**Published:** 2026-06-09

**Authors:** Amalia E. Yanni, Yiannis Kourkoutas

**Affiliations:** 1 Laboratory of Chemistry-Biochemistry-Physical Chemistry of Foods, Department of Nutrition and Dietetics, Harokopio University, 17671 Athens, Greece; 2 Laboratory of Applied Microbiology and Biotechnology, Department of Molecular Biology and Genetics, Democritus University of Thrace, 68100 Alexandroupolis, Greece; ikourkou@mbg.duth.gr

Food and its constituents influence human physiology and metabolism, thereby playing a crucial role in overall health. Owing to their potential to provide benefits beyond basic nutritional requirements, functional foods have attracted growing attention from researchers, consumers, and the food industry [1]. Experimental, clinical, and computational studies suggest that food-derived bioactive compounds may modulate metabolic outcomes through multi-target and system-level mechanisms, highlighting their potential as complementary strategies for supporting health maintenance and disease risk reduction [2,3].

In this vein, the second volume of the Special Issue of Nutrients, entitled “Functional Foods and Sustainable Health”, brings together a diverse collection of studies exploring emerging food-derived bioactive ingredients with strong potential for promoting human health. This volume highlights recent advances in the use of plant extracts [4], marine polysaccharides, probiotics [5], insects, microalgae, and novel sweeteners [6], all of which are being investigated for their roles in supporting disease prevention and management. Collectively, these contributions reflect the growing scientific interest in the development of innovative functional foods as part of strategies for improving health outcomes and advancing sustainable nutrition.

To start with, the systematic review by Kaim and Labus (Contribution 1), examined the physiological effects, health benefits, and regulatory considerations associated with monk fruit extract (MFE), as today there is a growing scientific interest in identifying natural, non-caloric sweeteners that can replace sugar without compromising taste or metabolic health [7]. This natural sweetener has gained attention due to its unique mogroside properties, which provide intense sweetness without rising glucose levels like traditional sugars. The findings, based on evidence from randomized controlled trials, indicated that MFE may positively influence glycemic control, reduce inflammatory responses, and support cardiovascular health. However, the current body of evidence is still limited, underscoring the need for long-term clinical trials to establish the safety and the potential role of MFE in chronic dietary interventions.

The review by Khandwal et al. (Contribution 2), comprehensively examined the diverse therapeutic potential of sulphated polysaccharides (SPs) derived from *Gracilaria* species, a widely distributed genus of red macroalgae, which have gathered significant attention. The work focused on the biological mechanisms of SPs and their role in mitigating chronic conditions, including their anti-cancer, anti-inflammatory, antioxidative, immunomodulatory, and antidiabetic effects, as well as neuroprotectant and prebiotic properties. Experimental studies in animal models have shown that these compounds can inhibit tumor growth and exert neuroprotective actions linked to reduction of oxidative stress. These polysaccharides have also demonstrated the ability to improve glucose and lipid metabolism and are capable of modulating immune function. Overall, these findings supported the potential use of *Gracilaria* SPs as functional food ingredients in the prevention and management of chronic diseases. However, further research is needed to confirm their safety, efficacy, and practical applications.

The neuroprotective potential of *Ocimum americanum* extract is presented in the study by Siqueira et al. (Contribution 3), which showed that antioxidant and anti-neuroinflammatory activities are the main properties counteracting the key-factors of neurodegenerative diseases, oxidative stress, and inflammation. The extract is rich in phenolic compounds, which contribute to antioxidant activity, while chronic supplementation lowers reactive oxygen species in the aged hippocampus. In addition, reduced cell membrane damage, mitochondrial protection, and lower concentrations of pro-inflammatory cytokines were observed. The study, which was conducted in rats, provided the first in vivo evidence supporting the potential application of *O. americanum* as a functional food ingredient aimed at reducing or delaying neurodegenerative changes associated with aging.

The study by Yokoyama et al. (Contribution 4) investigated the effects of the consumption of *Nopalea cochenilifera* (L.) *Salm-Dyck* cladodes on lipid metabolism, immune function, and gut microbiota in mice. The results demonstrated that supplementation of a high-fat diet with *N. cochenillifera*, which is known for their antioxidant and immunomodulatory effects, led to lower serum total cholesterol levels, improved intestinal barrier function, and beneficial alterations in the gut microbiota diversity. In particular, there was an increase in the abundance of butyrate-producing bacteria and a reduction in microbial populations linked to impaired lipid metabolism. Overall, the findings support the potential use of *N. cochenillifera* as a sustainable functional food ingredient for future applications.

The potential of combining probiotics with nutrient-rich food carriers to create innovative functional foods aimed at improving metabolic health and reducing inflammation in conditions like type 1 diabetes mellitus was investigated by Prapa et al. (Contribution 5). The findings of this study demonstrated that administering immobilized *Pediococcus acidilactici* SK cells on pistachio nuts to a diabetic animal model increased the abundance of lactic acid bacteria, which are associated with improved gut health and reduced the presence of opportunistic pathogenic microorganisms in fecal samples. The immobilized probiotic cells on pistachio nuts caused a more pronounced anti-inflammatory effect, as evidenced by lower levels of the pro-inflammatory cytokine IL-1β, suggesting that the delivery substrate (pistachio nuts) enhanced the functionality of probiotic cells, potentially due to improved stability or synergistic effects with the naturally occurring bioactive compounds in pistachio.

The study by Hu et al. (Contribution 6), investigated the effects of heat-killed *Lactobacillus delbrueckii* subsp. *lactis* 557 (LDL557) extracts on chondrocytes under osteoarthritic conditions. This in vitro cell culture study concluded that LDL557 extracts can preserve chondrocyte integrity and reduce cartilage degradation. By limiting inflammation-driven matrix breakdown, these extracts may contribute to maintaining joint health and slowing osteoarthritis progression. Consequently, LDL557 extracts emerge as a promising candidate for the development of alternative or complementary therapeutic strategies targeting this disease.

The potential of integrating edible insects and microalgae in conventional food products, such as baked goods, is underscored in the study by Zielińska et al. (Contribution 7). Despite their current perception as novel or unconventional within European dietary habits, these ingredients, when incorporated thoughtfully and in appropriate proportions, can enhance the nutritional profile of foods without compromising taste, texture or overall consumer acceptance. However, safety concerns regarding processing procedures and the risk of foodborne pathogen contamination, as well as the potential for allergic reactions to insect-derived foods, should be taken into serious consideration.

The studies featured in this Special Issue highlight the rapid expansion of functional food research, with a particular focus on natural bioactive compounds capable of modulating multiple pathways implicated in chronic disease development. To ensure their safety and facilitate their integration into everyday dietary patterns, further well-designed and rigorously controlled studies are required. Ultimately, these advances may support the development of functional foods that contribute to improved public health and the prevention of chronic diseases.
